# Paradoxes of Immunity: Alternative Forms of Adaptive Immunity Discovered by Evolutionary Genomics

**DOI:** 10.32607/actanaturae.27905

**Published:** 2026

**Authors:** E. S. Shilov, S. A. Nedospasov

**Affiliations:** Lomonosov Moscow State University, Faculty of Biology, Moscow, 119992 Russia; Engelhardt Institute of Molecular Biology, Russian Academy of Sciences, Moscow, 119991 Russia; A.N. Belozersky Research Institute of Physicochemical Biology, Lomonosov Moscow State University, Moscow, 119992 Russia

**Keywords:** evolution of adaptive immunity, fish, cyclostomes, MHC II, CD4, VLR

## Abstract

The fundamental discoveries in immunology in the 20th century initially focused on humans and other mammals, and then they extended to other vertebrates, including birds and fishes. Significant parallels in immune system architecture and mechanisms were revealed. In all organisms, from humans to zebrafish, antibodies and B cells, CD8^+^ and CD4^+^ T cells that recognize MHC I and MHC II, respectively, were identified, along with a set of major cytokines. Advances in DNA sequencing technologies and the development of bioinformatics for genome and transcriptome analysis allowed researchers to identify unique features in immune systems in some fishes and cyclostomes in the early 21st century. Such features stood in contradiction with established concepts based on studies of the mammalian immune system. The loss of several key genes encoding critical molecular mediators of adaptive immunity (MHC II, CD4, and others) was uncovered in anglerfish, codfish, and pipefish. Simultaneously, a unique type of adaptive immune system was discovered in lampreys and hagfish which utilizes different structural scaffolds (based on LRR-rich domains) to generate adaptive immune receptors (VLRs), as well as antibodies. Moreover, most species that could be classified as immunodeficient based on the studies of the vertebrate immune system proved to retain protective functions, suggesting the existence of compensatory mechanisms.

## INTRODUCTION

During the second half of the 20th century and the early 21st century discoveries and technologies based on laboratory mouse genetics, ranging from the establishment of homozygous inbred strains to the creation of thousands of transgenic and knockout mouse models, greatly facilitated our understanding of immune response mechanisms. The combination of these models allowed for the whole-organism assessment of the consequences of both regulated overexpression of immune regulatory proteins and the effects of complete or conditional deficiency of such proteins. Overall, the mouse and human immune systems are quite similar, consistent with the relatively conserved mechanisms of vertebrate immunity. Differences between mouse and human immunity reflect the lifestyle characteristics of rodents and primates and are explained primarily by changes in the levels and patterns of expression of the same set of genes. However, studies of the genomes of various vertebrates in the 21st century revealed examples of significant deviations from the classical general plan of the immune system structure, primarily the absence of individual key genes and regulatory cascades in the acquired immunity, which, according to previously prevailing ideas, should lead to significant defects in the mechanisms of immune defense [1]. Interestingly, in many cases, the consequences of a genetic deficiency of such immunoregulatory mechanisms proved to be much milder than the loss of function of the same genes in mice or humans. All this points to the existence of alternative immune mechanisms that compensate for the loss of the corresponding genes and proteins, or to a possible redistribution of functions between different components of the immune system. Of the approximately 65,000 species of living vertebrates, about 30,000 are bony fishes, about 1,000 are cartilaginous fishes, and about 100 species are cyclostomes. In particular, the immune system of the *Danio rerio* (striped zebrafish) has been well studied, with its full-fledged humoral and cellular immunity confirmed, and some new molecular mediators not typical for mammals found, such as NITR receptors on NK cells [2]. However, despite the general principles of the acquired immune system and the structural similarity of key molecules, a number of mechanisms are completely absent at the genetic level in certain groups of fish. This brief review examines several, unexpected aspects of the acquired immune system in fish.

## HOW ACQUIRED IMMUNITY WORKS

To emphasize the significance of gene loss, let us briefly recall the principles of acquired (adaptive) immunity in vertebrates. This aspect of immunity is based on a system of cellular receptors characterized by enormous diversity, as well as by distinct recognition principles that are not found in the innate immune system. For example, the major humoral factors related to acquired immunity are antibodies secreted by plasma cells, which represent the final stage of B cell differentiation. As a result of genomic rearrangements, functional genes encoding the heavy and light chains of antibodies are formed from multiple gene segments. First, in a maturing B cell, a sequence encoding the N-terminal variable domain of the heavy chain (designated V_H_) is assembled from three parts (V, D, J segments) through the process of V(D)J recombination [3]. The segment boundaries are marked by special oligonucleotides – RSS (recombination signal sequence), recognized by the specific recombinase RAG (recombination-activating genes) [4]. Then, using the same mechanism, a sequence encoding the N-terminal variable domain of the light chain (V_L_) is assembled from two segments (V and J). Either of the two types of light chains (κ and λ) can combine with any of the five types of heavy chains (μ, δ, α, γ, and ε). A naive (not yet having encountered its antigen) B lymphocyte can synthesize two types of heavy chains: μ and δ, with the same specificity due to alternative splicing, which selects a set of exons encoding conserved domains. The B cell receptor (BCR) is structurally equivalent to an antibody anchored to the B cell membrane and capable of transmitting an intracellular signal (e.g., proliferation or differentiation) in response to binding of the receptor to an antigen [5]. The BCR complex includes accessory proteins that transmit the intracellular signal: CD79a and CD79b [6]. After encountering an antigen, the B lymphocyte undergoes another rearrangement of its DNA, combining the sequence encoding V_H_ and exons encoding new types of heavy chains: α, γ, and ε. This process is called class switch recombination (CSR), the key event of which is the deamination of cytosine residues by the enzyme AID (activation induced deaminase) [7]. In addition, the sequences encoding the V_H_ and V_L_ domains undergo AID-mediated somatic hypermutagenesis during the development of the immune response in a special compartment located inside the secondary lymphoid organs – the germinal centers, leading to the accumulation of point mutations that allow for the selection of B lymphocytes with the highest affinity to their receptors, as well as that of soluble antibodies [8].

T cell receptor (TCR) diversity is also achieved through V(D)J recombination. As in the case of B cells, exons encoding variable domains are assembled in a RAG-dependent manner: the first assembled from three segments; and the second, from two segments. In this case, TCRs recognize and bind not free antigens, but rather epitopes (oligopeptides) loaded onto special MHC (major histocompatibility complex) molecules [9]. To transmit the signal, the TCR uses additional transmembrane proteins, collectively called the CD3 complex. There are two classes of MHC molecules: MHC I binds to and presents the oligopeptides of 8–11 amino acids from the cellular self-proteome, while MHC II binds to and presents longer peptides derived from proteins captured by endocytosis [10]. MHC II proteins are expressed on specialized antigen-presenting cells (APCs), which include macrophages, dendritic cells, and B lymphocytes. MHC II molecules interact with the TCR in a complex with the CD4 co-receptor protein, which is found on T helper (Th) and regulatory T cells (Treg); in this case, T cell activation leads to the synthesis of cytokines that regulate the immune response [11]. In contrast, MHC I molecules are present on almost all human cells, with the exception of erythrocytes, sperm, and eggs, and are necessary for the activation of T killers, which possess a heterodimeric CD8 co-receptor protein. Killer T cells recognize antigens from virus-infected or tumor cells and then trigger apoptosis in them either by injecting granzymes through the perforin pore or by stimulating the Fas receptor with the FasL ligand [12]. For the two polypeptides α and β to combine into an MHC II molecule, the CD74 protein (also called the “invariant chain”) is required; a fragment of this protein is initially inserted into the antigen-presenting cleft instead of the antigen peptide. During maturation in the thymus, T lymphocytes encounter epitopes from virtually any protein in the body, since, under the control of a special transcription factor, AIRE (and some others), the thymus epithelium expresses more than 95% of the genes in the genome [13]. If a T cell recognizes such an epitope in the thymus (i.e., is potentially autoimmune), it either dies via apoptosis or differentiates into a suppressor regulatory T cell (Treg), which will subsequently suppress immune responses directed against this epitope [14]. In general, this architecture of adaptive immunity is characteristic of most vertebrates, starting with cartilaginous fish. The emergence and complexity of the immune system during vertebrate evolution is shown in *Fig. 1.* For a more detailed overview of the early evolution of acquired immunity reviews [15, 16] can be recommended. Interestingly, in terms of the number of evolutionary acquisitions, the immune system of birds is more advanced than that of mammals. Avian B cells mature in a unique primary lymphoid organ – bursa of Fabricius, where a fully developed secondary repertoire is created through AID-mediated gene conversion based on a very limited primary antibody repertoire (created through V(D)J recombination). Although many mammals (but not humans or mice) also use gene conversion to expand their antibody repertoires, they have not developed new lymphoid organs.

**Fig. 1:**
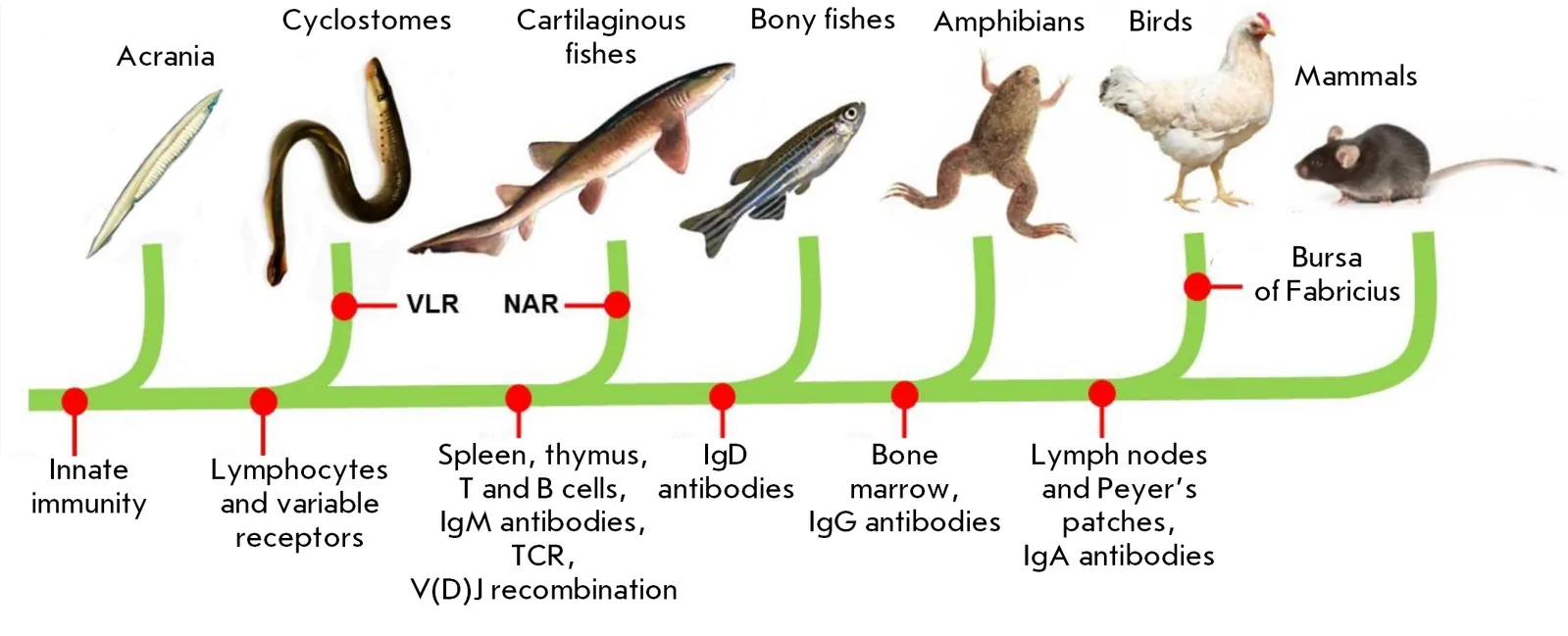
The emergence and complexity of the vertebrate immune system during evolution. The lancelet (an acrania chordate) lacks elements of acquired immunity, just like the common ancestor of vertebrates. Red dots on the evolutionary tree represent the emergence of important structural and functional organs, molecules, and processes

One of the central paradigms in our knowledge of vertebrate adaptive immunity is the T helper cell concept, which is based on the fact that without functioning T cells, the B cell response is defective. This concept has subsequently been expanded, and four main types of T helper cells are now distinguished, with only one of them (Tfh) directly involved in “helping” B cells. Importantly, all four of these helper cell types are CD4^+^ T cells, whose TCR recognizes peptide antigens in complex with MHC II molecules. Finally, there exists a special population of immunosuppressive T cells called regulatory T cells (Treg) which also express CD4 [17]. The importance of this cell population was highlighted by the 2025 Nobel Prize. It turned out that the possibility of transdifferentiation of T helpers into regulatory T cells is determined by the regulatory sequence (enhancer) CNS1 of the *Foxp3* gene, which is characteristic only of placental mammals and arose as a result of retrotransposition [18]. Deletion of the CNS1 enhancer reduces the proportion of viable embryos; thus, the complication of the regulation of the *Foxp3* gene and the emergence of peripheral Tregs has become one of the key adaptation paths to viviparity in mammals. Deficiency of Tregs leads to impaired immunological tolerance, activation of autoreactive lymphocytes clones and, ultimately, to multiple autoimmune pathologies, as found in mice with the *scurfy* mutation*.* A diagram briefly showing the principles of adaptive immunity and the key proteins of lymphocytes is presented in *Fig. 2.* It should be noted that T helpers are not a unique population capable of controlling the immune response by synthesizing cytokines: in vertebrates, there are also three types of lymphoid cells of the innate immune system (ILC 1–3) corresponding to the profile of secreted cytokines to the main populations of T helpers [19]. In addition, it should be taken into account that B-lymphocytes can also secrete a variety of cytokines during the immune response, including immunosuppressive IL-10, IL-35, β-TGF, and proinflammatory IL-6, IL-12, and TNF [20]. B lymphocytes secreting IL-10 were named regulatory B cells (Breg) in 2002 [21]. Interestingly, even some CD8-positive T cells are capable of acting as T helpers, simultaneously providing both the secretion of various cytokines and contact stimulation via CD40L [22].

**Fig. 2:**
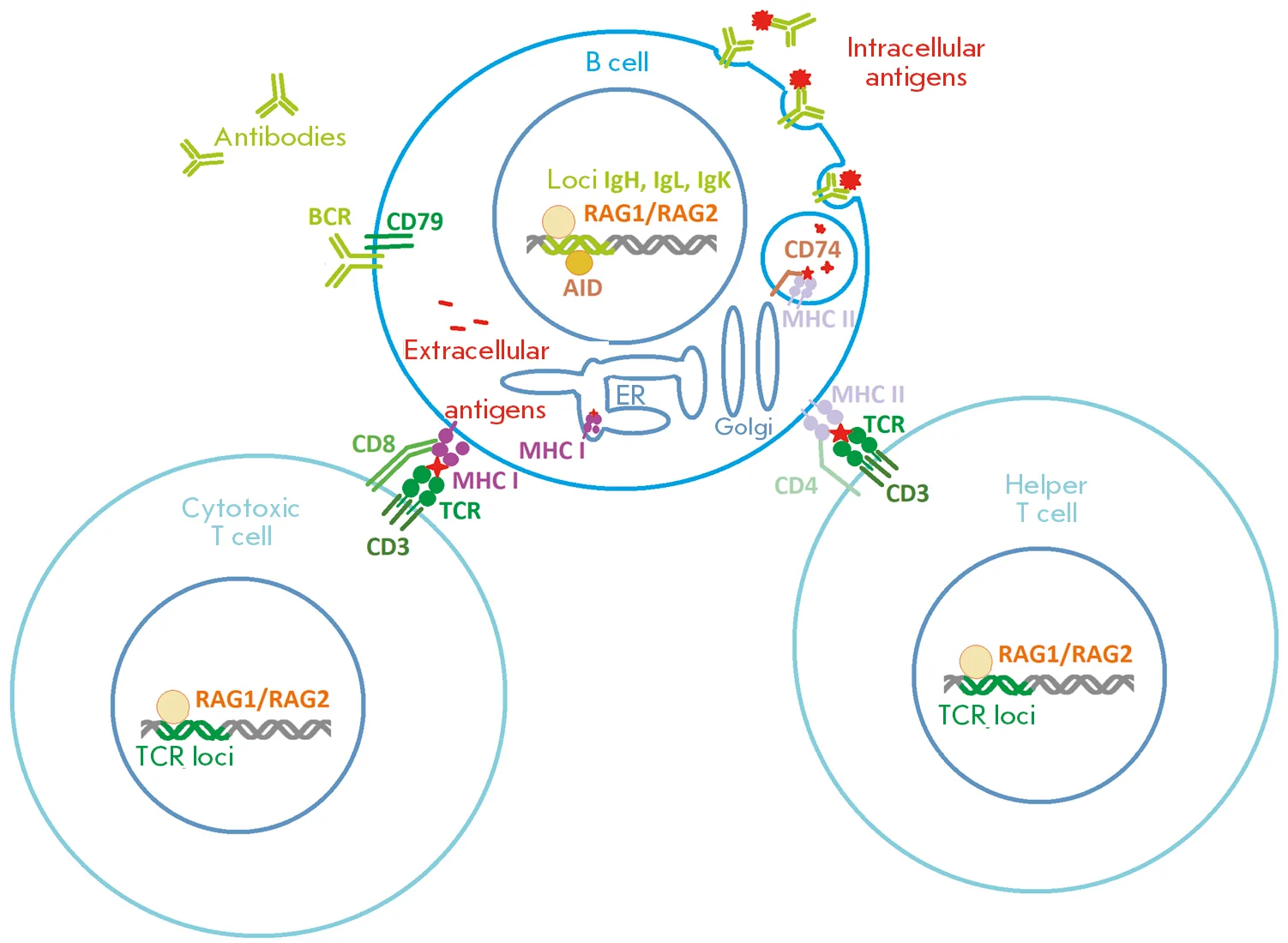
The key proteins of adaptive immunity. The B cell was chosen as the antigen-presenting cell, which after costimulation by a T helper cell through CD40 molecule differentiates into a plasma cell secreting antibodies. Recognition of antigens by the BCR receptor and presentation of intracellular antigens using MHC are demonstrated. I and presentation of extracellular antigens by MHC II

By the close of the 20th century, technologies of reverse genetics made it possible to create mice lacking putative key genes of the vertebrate immune system and to determine the phenotypes of such mice. Specifically, mice were created with genetic knockouts of the recombinase complex genes (RAG1/RAG2), which regulates the rearrangement of immunoglobulin gene loci and is essential for the vast diversity of T and B cell receptors, a key feature of adaptive immunity [23]. These mice are profoundly immunodeficient, as they do not undergo proper differentiation of T and B lymphocytes. However, they are viable, have a normal lifespan in SPF animal facilities (of course, they would not be able to survive in the wild), and have become highly valuable models for immunobiological research. In addition (and this is important for our subsequent discussion), separate knockouts of the T-lymphocyte coreceptors CD4 [24] and CD8 [25] were generated and mice with a deletion of MHC class II genes were created [26]. These mice also showed defects in immunoregulation and host defense functions, especially against viral infections, but these defects were not as profound as mice with RAG1/2 knockouts. The absence of one coreceptor leads to a redistribution of lymphocytes in favor of the other; for example, CD8 knockout mice that retained CD4 were found to be more resistant to the trypanosome *Trypanosoma brucei* than CD4 knockout mice and wild-type mice [27]. This natural compensation occurs because the thymocyte chooses which of the two MHC types better recognizes its TCR – if one of the coreceptors is missing, all surviving T cells differentiate in the only available direction. Genetic knockouts of individual genes encoding the CD3 [28, 29] and CD79 [30] receptor proteins result in the cessation of T-cell and B-cell development, respectively. Knockout of CD74 (MHC invariant chain) disrupts MHC II assembly and B-cell maturation [31]. Knockout of AID results in impaired antibody class switching by B cells and inhibition of somatic hypermutagenesis, with the only secreted antibodies being IgM [8]*. Foxp3* knockout mice recapitulated the phenotype of *scurfy* mutant mice [32]. Sometimes genetic knockouts result in obvious morphological changes in the immune system. For example, *Lta* knockout mice lack lymph nodes and Peyer’s patches and fail to form germinal centers in the spleen, which are important for antibody affinity maturation [33, 34]. Knockouts of genes for other members of the TNF family also exhibit morphological manifestations: knockout of the *Tnf* gene leads to the loss of Peyer’s patches and germinal centers [35]. Mice with triple knockout *of Tnf*, *Lta*, and *Ltb* [36] lack lymph nodes, Peyer’s patches, and germinal centers in the spleen; and *Tnf* and *Ltb* double knockout mice lack Peyer’s patches and germinal centers [37]. It is interesting to note that the phenotype of knockout mice depends not simply on the presence or absence of a particular cytokine in the body, but also on whether this cytokine is produced by certain cells of the immune system. For example, among several conditional knockouts of the *Tnf* gene analyzed, loss of Peyer’s patches is observed only in innate immune lymphoid cell knockouts of IL3, whereas Peyer’s patches are retained in T-cell and myeloid knockouts [38].

## LOSS OF KEY GENES OF ACQUIRED IMMUNITY IN FISH

In general, the immune system of cartilaginous and bony fishes is structured somewhat differently from that of birds or mammals [15]. Fishes have a thymus (a paired organ in the walls of the gill cavities [39]) and a spleen, but no red bone marrow; instead, hematopoiesis occurs in the kidneys and other organs. They lack lymph nodes and germinal centers in the B-cell zone of the spleen. Although somatic hypermutagenesis occurs in B-lymphocytes, isotype switching by the CSR mechanism does not occur and at least three types of antibody light chains have been found: κ, λ, and σ (a fourth type, σ2, is sometimes added). Cartilaginous fishes have three types of heavy chains, corresponding to IgM, IgW, and IgNAR antibodies, and bony fishes also have three types of heavy chains, corresponding to IgM, IgD, and IgT antibodies in [15]. These differences between fish and mammals should be kept in mind when further discussing the loss of a number of immune system genes.

The first, rather sensational example of the loss of important immune components in fish was the complete absence of the MHC II and *CD74* genes, as well as the transformation of the *CD4* gene into a nonfunctional pseudogene in the cod *Gadus morhua*, discovered in 2011 as a result of whole-genome sequencing [40]. At the same time, the cod genome showed a significant increase in the number of genes encoding innate immunity receptors from the TLR family, as well as MHC I genes. In this regard, a hypothesis has been put forward according to which MHC I in cod can functionally replace MHC II, assuming a mechanism similar to the cross-presentation of external antigens in mammals. There is some evidence supporting this idea, as translocation of MHC I proteins and the chaperones that service them from endoplasmic reticulum (ER) to endosomes was demonstrated, thus implying a possible loading of extracellular antigens into MHC I [41, 42]. Interestingly, the expansion of MHC I genes in cod was noticed as early as 1999 during the analysis of cDNA sequences [43]. Additionally, as experimentally demonstrated in 2022, cod AID deaminase lacks catalytic activity; i.e., it cannot ensure somatic hypermutagenesis in B cells [44]. This observation is in agreement with an earlier report on the failure to generate cod antigen-specific antibodies during standard immunization with an antigen in complete Freund’s adjuvant [45], which led to the hypothesis of defective MHC II functions in cod [46]. Nevertheless, the humoral immunity of cod appears to be fully functional, as the presence of full-fledged tetrameric (characteristic for fish) IgM immunoglobulins in blood plasma was demonstrated as early as 1991 [47]. Immunization of cod results in the formation of antigen-specific IgM antibodies [48], which enable fish to defend themselves against the pathogenic bacterium *Vibrio anguillarum* if a vaccine based on formalin-inactivated bacteria is used [49]. Apparently, the lack of somatic hypermutagenesis and the absence of T-helpers in cod are somehow compensated for by an excess of TLRs, cross-presentation of external antigens on MHC I, and high-affinity IgM antibodies formed independently of T-cell help. Which cells in the cod immune system lacking CD4 take over the functions of Treg remains an open question. To date, there have been no reports of cod cells expressing Treg-specific markers, including the transcription factor Foxp3. It is quite possible that such suppressor cells may be found among CD8-positive T cells.

The absence of MHC II genes was also shown in many other fishes. For example, the common ancestor of fishes from the order *Gadidae* lost the MHC II genes during the evolution of ray-finned fishes approximately 95 million years ago, while the common ancestor of cods and sunfishes lost the *Mx* gene (encoding a protein for resistance to myxoviruses) approximately 120 million years ago, and the common ancestor of cods, sunfishes, and polymyxids lost the flagellin receptor TLR5 150 million years ago [50]. Random deletions occurring either as a result of genetic drift or natural selection aimed at reducing the consumption of metabolic resources by the immune system [51] have been suggested as the cause of the loss of MHC II. In a 2013 study, neither the MHC II, CD4, or CD8b genes were found in the transcriptome of the longnose pipefish *Syngnathus typhle* [52]. However, other fishes from the same order, the Echinodermata, did possess these genes, limiting the list of immunodeficient fish to seahorses of the genus *Hippocampus* and pipefish of the genus *Syngnathus* [53]. A common feature of these two groups of fish is “male pregnancy”, the implantation of fertilized eggs into a specialized brood pouch in males which possesses a placenta-like structure. Therefore, it was suggested that the loss of MHC II and a number of other changes in the genes of acquired immunity allow seahorses and pipefish to carry their eggs without the risk of rejection [53]. However, these data should be treated with caution, because the transcriptome analysis, unlike whole genome analysis, does not indicate a reliably proven absence of any specific gene. In 2016, sequence analysis of seahorse *H. erectus* genes for the α- and β-chains of MHC II (only one gene of each type, which is not typical for vertebrates) showed a typical allelic diversity for these genes [54]. However, the same group that published data on the absence of MHC II in seahorses reported in 2024 an increase in the expression of MHC II genes in the tissues of *H. erectus* in response to immunization with heat-killed bacteria [55], consistent with the classical immunological paradigm of T cell activation by activated antigen-presenting cells.

Interestingly, significant evolutionary immunodeficiency can develop without the complete loss of MHC genes. For example, in the genome of the “living fossil” coelacanth, no locus for the heavy μ-chain of IgM antibodies was found but two loci corresponding to IgD/IgW antibodies were present [56]. Fish from the order Anglerfish have gone the furthest along the path of immune gene loss. In 2020, a substantiated hypothesis was put forward positing that differences in the immunity of anglerfish may be associated with differences in their reproductive strategy [57]. There are three main types of reproductive strategy in anglerfish: 1) mating between an independent male and female of approximately the same size, 2) mating of a miniature parasitic male temporarily attached to a large female, and 3) mating of a large female with one or more parasitic dwarf males that are permanently attached to and fused with her body. In the first case, anglerfish retain a full set of genes for acquired immunity; in the second, they lose the AID deaminase gene (and probably the ability for somatic hypermutagenesis of antibodies); in the third case, the AID genes are lost, as well as those for CD3 and CD8 proteins, and in some species, the genes for RAG recombinases, CD4, as well as the B cell receptor protein CD79 and the CD74 protein, which are necessary for the assembly of MHC II, are additionally lost [57]. The European anglerfish *Lophius piscatorius* has lost the MHC II, CD4, and CD74 genes, despite the fact that it does not have sexual dimorphism and parasitic males [58]. However, the MHC I, CD3, and CD8 genes are retained in the European anglerfish.

Apparently, the loss of adaptive immunity genes conferred on anglerfish certain advantages, such as the colonization of a luminous bait organ (esca) with luminescent bacteria or the parasitism of dwarf males on females. Moreover, this process could have proceeded in parallel in several evolutionary lineages of anglerfish. A study of the genomes of a large number of anglerfish showed that the gradual loss of adaptive immunity genes occurred against the background of the transition from a benthic to a pelagic lifestyle (in the water column), with a simultaneous decrease in the size of males [59]. Moreover, the attachment of dwarf males to females, at first temporary, for mating, and then permanent, became a key adaptation that allowed anglerfish to master the deep sea [60, 61]. Apparently, given the low probability of meeting a partner at a time convenient for reproduction, the lack of an acquired immune response became an acceptable price for anglerfish in exchange for a reliable reproductive strategy. However, given the loss of immune system genes in anglerfishes without sexual dimorphism, such as *Lophius piscatorius*, it is likely that the initial stimulus for such a loss was the need to colonize the *esca* (bait organ) with luminescent bacteria from the environment. Analysis of the microbiomes of fry and adult anglerfish revealed their species-specificity and significant differences between fry and adults, with enrichment of luminescent bacteria (*Enterovibrio luxaltus* and others) specific to the esca of adult fish [62]. For successful colonization of luminescent organs by luminescent bacteria, fish may benefit from the loss of humoral immunity, for example, due to the loss of AID deaminase or the B cell receptor protein CD79. If this hypothesis is correct, then the loss of immune system genes should be expected in other deep-sea fishes with bioluminescent organs, such as chauliods, bathophils, garganeys, luminescent anchovies, and others. Their genomes have not yet been analyzed.

Australian ghost shark *Callorhinchus milii* (which is not a true shark, but a representative of chimeras, another order of cartilaginous fishes) has MHC genes but lacks a functional CD4, as well as the transcription factors RORC and Foxp3, which are necessary for the development of Th17 and Treg, respectively [63]. Venkatesh et al. suggested that this represents an intermediate evolutionary state characteristic of all cartilaginous fishes. However, Takizawa et al. believe that cartilaginous fishes, such as the Japanese catshark *Scyliorhinus torazame* and the nurse shark *Ginglymostoma cirratum*, possess functional CD4, and that the gene described in [63] as CD4-like is a full-fledged CD4 [64]. In general, this point of view seems more substantiated. The losses of individual components of immunity in different groups of fish during evolution are summarized in *Fig. 3.*

**Fig. 3:**
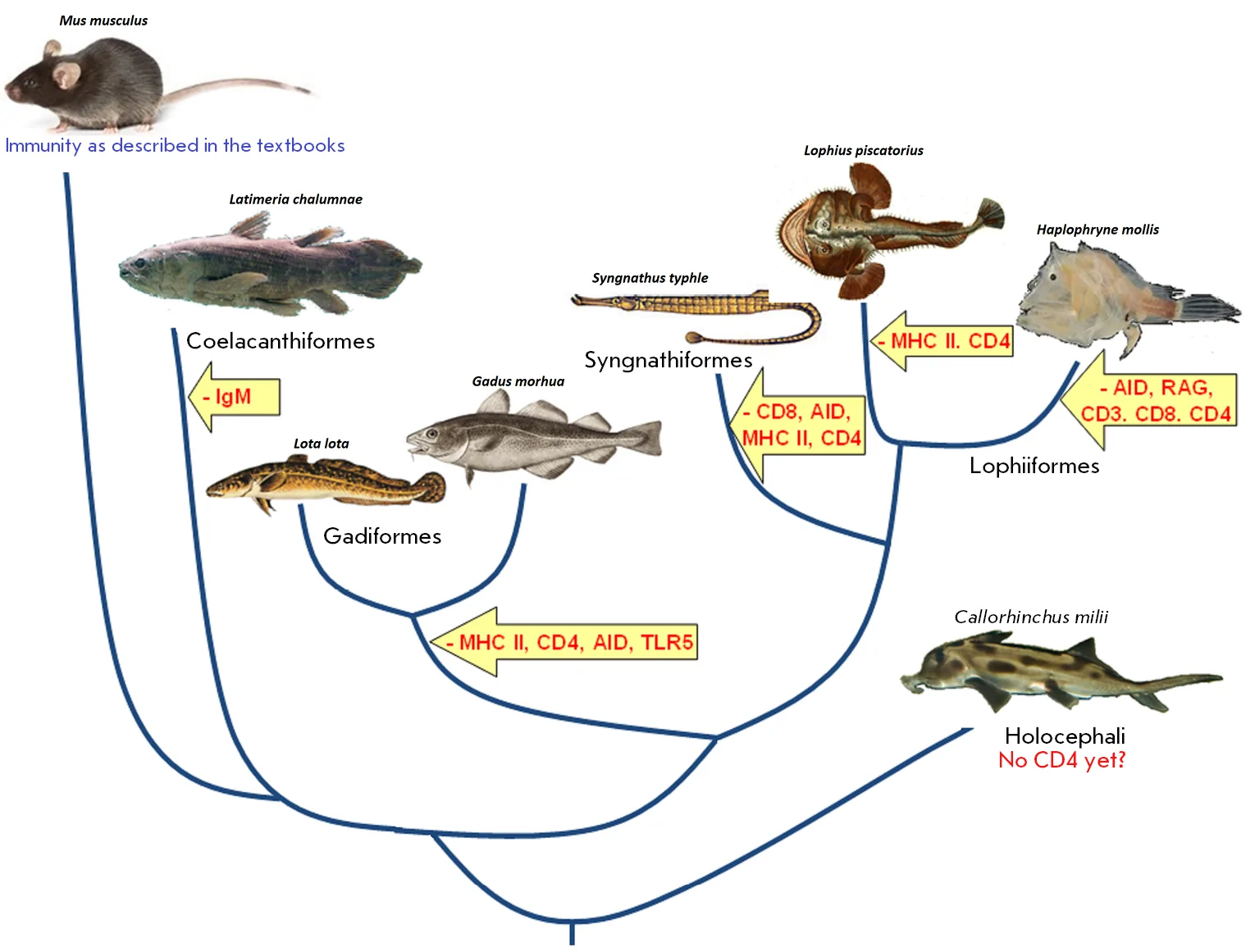
The loss of key immune proteins in fish. Only those groups are shown whose members, according to current data, have lost genes encoding the individual key proteins involved in the immune response. When multiple proteins are listed, the arrow indicating the loss corresponds to several successive evolutionary events

## THE IMMUNITY OF CYCLOSTOMES

In 2004, jawless vertebrates (lampreys and hagfish) were found to possess a unique, acquired immunity system that recognizes antigens using variable lymphocyte receptors (VLRs) [65]. This discovery was made through a bioinformatics analysis of the transcriptome of activated lymphocytes from lamprey larvae. VLRs proved structurally similar to the Toll-like receptors of innate immunity, with rigidity provided by short parallel beta-sheets that are part of the leucine-rich repeats (LRRs) and retention on the plasma membrane by a GPI anchor [65]. Analysis of the structure of VLR monomers revealed a horseshoe-shaped structure that binds antigens with a variable concave surface, precisely in the region of the beta-sheets. Variable lymphocyte receptor (VLR) genes are absent from the native cyclostomal genome and are assembled through gene conversion in developing lymphocytes using donor cassettes of leucine-rich repeat-encoding sequences. In lampreys (hagfishes were much less studied), six types of VLR receptors have been found to date: VLRA, VLRB, VLRC, VLRD, VLRE, and VLRF [66]. Lymphocytes expressing VLRBs are functional analogues of B cells, whereas lymphocytes expressing the other five VLR types are more similar to T cells. VLRB^+^ lymphocytes have two forms of variable receptors: membrane-bound (via a GPI anchor) and secretory [67]. Secretory VLRBs are oligomeric, comprising 8–10 identical subunits linked by disulfide bridges; they are capable of initiating the VLRB-mediated complement activation pathway [68]. Analogues of vertebrate FcR receptors that bind antibodies were not found in cyclostomes. VLRA^+^, VLRC^+^, VLRD^+^, VLRE^+^, and VLRF^+^ lymphocytes are likely to be functionally more similar to γδ T cells than to gnathostome αβ T cells, since they bind antigens directly, without presenting peptides on MHC molecules (which cyclostomes lack) [69].

The mechanism for generating VLR diversity in cyclostomes differs fundamentally from V(D)J-recombination in other vertebrates, but it resembles the process of antibody isotype switching. Cyclostome genomes contain *VLR* gene templates in which noncoding spacers are located between the N- and C-termini of the VLR-encoding sequences; the *VLRB* template also contains a region from the middle of the coding sequence. During lymphocyte maturation, the spacers are replaced through several successive rearrangements by coding sequences fragments borrowed from special cassettes located in the same VLR loci [70]. Strictly speaking, the term “cassette” is not entirely applicable in this case, since these sets of short coding sequences have neither a defined order, nor distinct boundaries, like the RSS sequences of vertebrates. “T cell” lymphocytes of cyclostomes mature in the thymoids – special areas of the gill filaments, whereas VLRB^+^ lymphocytes develop in the kidneys and the intestinal outgrowth called the typhlosole. In the lymphoid organs of cyclostomes, VLR-positive lymphocytes express the cytidine deaminases CDA1 and CDA2 from the AID/APOBEC family. It is assumed that these enzymes deaminate cytidine nucleotides within the VLR loci, converting them into uridine, after which the DNA repair system removes such nucleotides and rejoins homologous sequences that were originally located in different regions of DNA [71]. The VLR system of the sea lamprey *Petromyzon marinus* has been the most thoroughly studied. The lamprey has a genome with a total size of approximately 2 billion bp, divided into 84 chromosomes. The VLRC locus in this species is located on chromosome 7; VLRB, on chromosome 21; VLRA and VLRF, on chromosome 58; and VLRD and VLRE, on chromosome 75. Interestingly, the VLRA, VLRC, VLRD, VLRE, and VLRF receptor genes share common segment donor cassettes through intrachromosomal and even interchromosomal rearrangements, while VLRB uses a separate, specialized set of segments [66, 72]. During maturation, cyclostomal T-lymphocyte analogues can express several different types of “T cell” VLRs, but mature cells apparently express only one type of VLR with a particular specificity. How allelic exclusion is maintained remains unknown. Lymphocyte selection is aimed at producing a VLR of a certain length and, probably, at interaction with a not-yet-described protein partner [73]. A similarly structured system of VLR receptors and VLR-positive cells is also found in hagfish [74], but the number of described types is limited to three: VLRA, VLRB, and VLRC [66]. A schematic representation of the immune system of the hagfish is shown in *Fig. 4.* At the same time, lampreys change their diet during metamorphosis and significantly alter the anatomy of their respiratory and digestive systems (for example, the typhlosole is absent in sexually mature lampreys). It should be noted that the immune system of sexually mature lampreys has not been sufficiently studied.

**Fig. 4:**
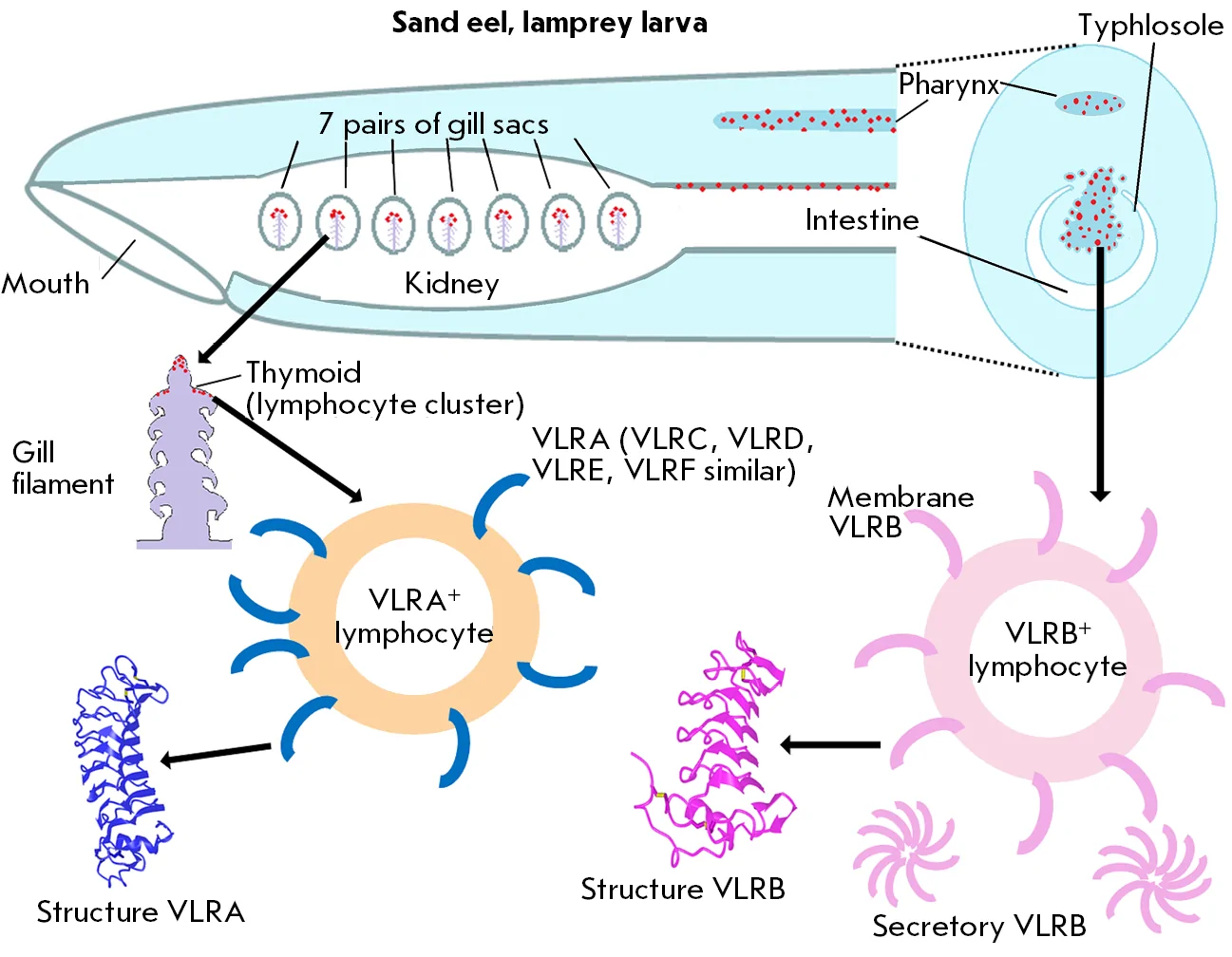
The lymphocytes and primary lymphoid organs of lampreys. The head end of the body and a cross section (at the level of the kidney) of the sand eel are shown. The development of the “T cell” lymphocytes VLRA^+^, VLRC^+^, VLRD^+^, VLRE^+^, and VLRF^+^ occurs in the gills, in special zones of the gill filaments called thymoids, and the rearrangement of loci occurs with the participation of deaminase CDA1. VLRs bind antigens with their surface, marked with a black arrow. The development of “B cell” lymphocytes occurs in the hematopoietic zones of the intestine (typhlosole) and kidney, and the rearrangement of loci occurs with the participation of deaminase CDA2. Subsequently, any mature VLR lymphocyte can be found in the bloodstream and in peripheral tissues. The spleen, lymph nodes, or other secondary lymphoid organs are absent, as is the lymphatic system

In summary, the immune systems of gnathostomes and cyclostomes differ substantially. Despite a certain degree of complexity in cyclostomes (five different types of “T cells”, assembly of a full-length VLR coding sequence from a constellation of individual short sequences), their immune system is fundamentally simpler. Cyclostomes lack MHC molecules, antigen processing and presentation, and V(D)J recombination. There are no FcR analogues, and it is unknown how transplant immunity is achieved; a rather limited set of cytokines have been predicted: IL-4, IL-6, IL-7, IL-8, IL-10, IL-12, IL-16, IL-17, IL-35, as well as type 1 and 2 interferons [75]. Humoral immunity based on VLRB, apparently, is functionally comparable to humoral immunity based on antibodies, but a full-fledged analogue of T-cell immunity does not exist in cyclostomes. Cyclostome T cell lymphocytes (VLRA^+^, VLRC^+^, VLRD^+^, VLRE^+^, VLRF^+)^ are analogous to the γδ T cells of other vertebrates. The VLR membrane receptor is monovalent; it binds to its antigen in monomeric form. Further intracellular signal transduction likely requires VLR clustering and phosphorylation of proteins similar to CD3 and CD79; however, such proteins have not yet been identified. The differences in the immune system between cyclostomes and gnathostomes are summarized in *Table 1.*

**Table 1:** The differences in the immunity of cyclostomes and gnathostomes

Features of immunity	Cyclostomes	Gnathostomata
The mechanism of reorganization of variable receptor genes	CDA-mediated gene conversion	RAG-mediated V(D)J recombination, sometimes followed by conversion
The number of rearranged loci in the genome	Six loci: VLRA, VLRB, VLRC, VLRD, VLRE, VLRF	At least seven loci: IGH, IGL, IGK, TRB, TRG, TRA/TRD
Secreted variable molecule	VRB of 8–10 subunits	Pentameric IgM and other classes of antibodies
Antigen presentation to lymphocytes	Absent	On MHC I and MHC II molecules
Transplantation immunity	Moderately expressed, mechanism unclear	Strong, MHC-mediated
Interaction of innate and acquired immunity	Unknown	Underlies the activation of acquired immunity

## CONCLUSION

This brief review presents “stubborn facts” about the structure of the fish immune system (primarily the characteristics of their acquired immunity) that directly contradict the descriptions found in classical immunology textbooks. The main surprise is the existence of quite successful organisms (such as cod fish) that lack CD4^+^ T cells (since there is no functional CD4 co-receptor gene) in which the MHC class II genetic locus is reduced. Does this mean that they can live and provide antibacterial and antiviral protection without recourse to T helpers and regulatory T cells at all? Or have some other cells assumed the lost functions? Apparently, the expansion of MHC I genes [43] and the transition of MHC I proteins to the endosomal compartment [41, 42] indicate that CD8^+^ T lymphocytes of cod fish may take over some of the functions of CD4^+^ cells. There is no direct evidence for this at the moment; however, given the presence in mammals of subpopulations of CD8^+^ T cells (Tc1–Tc22) expressing CD40L and the main groups of cytokines [22, 76], a complete replacement of CD4^+^ T cells loss in fish appears quite plausible. However, the evolutionary reasons for the loss of MHC class II in codfish are not obvious; the scenario of a gradual loss of first Mx, then TLR5, and finally MHC class II [50] is based on the assumption of a fixation of deletions during random genetic drift in small populations. This selective loss of MHC class II genes while MHC class I genes are maintained is facilitated by the fact that in bony fishes, unlike most other vertebrates, these genes are located in two separate loci [15].

The hypothesis proposed by Star and Jentoft [51] concerning the adaptive significance of the loss of MHC II in cod fishes, associated with the economy of metabolic resources, facilitation of interaction with symbiotic microorganisms and other possible explanations, seems unlikely in this case. The example of anglerfish and pipefish shows how significantly such adaptive pressures affect the biology of fishes. Colonization of the esca by luminescent bacteria (anglerfish, including the European anglerfish), attachment of dwarf males to females (deep-sea anglerfish), and the incubation of fertilized eggs by males in a brood pouch (pipefish, seahorses) all appear to be significant factors favoring the disruption of the immune system through natural selection. Probably, in the future, the loss of immune system genes, also caused by close symbiosis with microorganisms, will be discovered in other luminous fishes. A loss associated with reproductive characteristics is found in other viviparous fishes and amphibians, including viviparous sharks, platies, and the Surinam pipa. Interestingly, the coelacanth, which exhibits a loss of IgM antibodies, is also viviparous [77]. However, the biology of cod, burbot, pollock, saithe, and other cod-like fishes does not present any striking features, which makes the state of their immune systems all the more surprising.

Another sensation was the discovery of a special form of acquired immunity in cyclostomes which is somewhat reminiscent of the immune system of vertebrates but uses different structural modules (LRR folds instead of Ig folds) as “scaffolds” for T and B cell receptors. It is obvious that the VLRB antibodies of cyclostomes are also constructed from such modules. Overall, the immune system of cyclostomes resembles the immune system of vertebrates but in cyclostomes all T cells are represented by γδ T cells and all B cells are represented by T-independent B1 lymphocytes. This echoes the ideas of Charles Janeway that in evolution γδ T cells were the first type of lymphocytes with variable receptors [78, 79]. One can speculate that jawed vertebrates with such a state of the immune system became extinct, but in cyclostomes, in a parallel line of evolution with a different type of variable receptors, this archaic architecture has been preserved.

Interestingly, the loss of individual genetic elements can subsequently lead to greater complexity of the immune system. Thus, deletions of exons encoding the first constant domain (C_H_1) of the heavy chain of IgG2 and IgG3 in camelids (camels, alpacas, llamas) led to the emergence of a new class of mammalian antibodies that do not have light chains – HCAb (Heavy-Chain only antibodies) [80]. In this case, IgG1 antibodies retained a full-length format and the emergence of HCAb as a result of the loss of C_H_1 occurred at least 3 times. The variable domain of HCAb antibodies is called V_H_H, it binds antigens without the help of additional proteins (single-domain binding), and it possesses unique structural features, such as an elongated hypervariable loop CDR3 and additional disulfide bonds between hypervariable loops [81]. Immunology textbooks continue to teach that a combination of variable domains of heavy and light chains is critical for high-affinity interaction with an antigen. The HCAb antibodies of lysopoda, discovered in 1993 [82], present an example of convergent evolution with IgNAR shark antibodies, discovered in 1995 [83], which also consist only of heavy chains. According to current concepts, IgNARs result from the loss of the first constant domain of the heavy chain of shark IgW antibodies [84]. Significant simplifications at the genomic level occur at the loci encoding the variable domains of antibodies. For example, birds lack the V- and J-segment cassettes of the heavy chain and the single V-segment is combined with one of the 15 D-segments and the only J-segment [85], while a completely uncontested rearrangement is characteristic of the V- and J-segments of the light chain [86]. Antibody diversity appears at a later stage of B cell development, within the bursa of Fabricius, due to AID-mediated gene conversion involving the pseudogenes of V-segments adjacent to immunoglobulin loci [87]. At the same time, the canonical structure of the TCR loci and full-fledged V(D)J recombination are preserved in avian T cells.
